# Heterologous Expression of Recombinant Ginseng Tetradecapeptide in *Saccharomyces cerevisiae* and Evaluation of Its Biological Activity

**DOI:** 10.3390/foods14122049

**Published:** 2025-06-10

**Authors:** Yi Qi, Pei Ma, Pan Wang, Chenhui Zhu

**Affiliations:** 1 Engineering Research Center of Western Resource Innovation Medicine Green Manufacturing, Ministry·of Education, School of Chemical Engineering, Northwest University, Xi’an 710127, China; 18049524478@163.com (Y.Q.); maipei@nwu.edu.cn (P.M.); 2 Shaanxi Key Laboratory of Biomaterials and Synthetic Biology, Shaanxi R&D Center of Biomaterials and Fermentation Engineering, School of Chemical Engineering, Northwest University, Xi’an 710127, China; 3 Biotechnology & Biomedicine Research Institute, Northwest University, Xi’an 710127, China; 4 Shaanxi Green Bio-Manufacturing Future Industry Research Institute, School of Chemical Engineering, Northwest University, Xi’an 710127, China

**Keywords:** *Saccharomyces cerevisiae*, ginseng peptides, multicopy tandem expression, diabetes mellitus

## Abstract

Ginseng peptides, as bioactive components of ginseng, have attracted increasing attention. In this study, a 14-amino acid ginseng peptide was selected and heterologously expressed in *Saccharomyces cerevisiae* using a multicopy tandem fusion strategy, named 7RS14α. The secondary structure of the recombinant ginseng tetradecapeptide (7RS14α) was analyzed, and a high-glucose model was established in mouse adipocytes to evaluate its biological activity. Transcriptomic profiling was further performed to elucidate its potential mechanisms. Results demonstrated that 7RS14α significantly enhanced glucose uptake in high-glucose model cells, likely by modulating lipid metabolism pathways and insulin signaling cascades, thereby influencing energy homeostasis in adipocytes.

## 1. Introduction

Diabetes mellitus is a chronic metabolic disease, characterized by persistent hyperglycemia resulting from defects in insulin secretion or action, and has emerged as a major global health threat [1]. It triggers multisystem complications, including diabetic ketoacidosis, vascular dysfunction, and neuropathy. Current therapeutic strategies for diabetes primarily focus on lifestyle interventions, pharmacological treatments (e.g., insulin analogs, metformin), and emerging approaches such as complementary and alternative medicine (CAM) [2]. However, these existing methods often fall short in effectively managing diabetes due to issues like drug dependency, side effects, and incomplete control of blood glucose levels [3]. This highlights the urgent need for novel therapeutic agents and innovative approaches to address the growing burden of diabetes [4].

In the quest for new treatments, natural products have garnered significant attention for their rich content of bioactive components and therapeutic benefits [5]. Hypoglycemic peptides, derived from natural or synthetic bioactive peptides with anti-hyperglycemic properties, are gaining attention as adjunctive therapies due to their low toxicity and multitarget regulatory mechanisms [6]. These peptides, derived from natural sources, such as soybean and bitter melon, or synthesized through various platforms, modulate glucose homeostasis by inhibiting digestive enzymes, mimicking insulin activity, or regulating gut hormone secretion [7]. In 2023, the global market for hypoglycemic peptides took shape, highlighting their clinical and commercial significance in reducing drug dependence and improving the quality of life of diabetic patients [8]. Among a wide variety of natural products, Panax ginseng C.A. Meyer, as a highly regarded medicinal plant, holds significant value for research and application [9]. Documented in classical texts such as Shennong Bencaojing (The Divine Farmer’s Materia Medica) and Huangdi Neijing (The Yellow Emperor’s Inner Canon), ginseng has been used for millennia to enhance recovery, stimulate blood circulation, alleviate stress, and combat fatigue [10].

Modern phytochemical studies have identified over 200 bioactive compounds in ginseng, including triterpenes, saponins (ginsenosides), alkaloids, peptidoglycans, and proteins. While ginsenosides have traditionally been recognized as their primary active constituents, recent research has uncovered the untapped potential of ginseng-derived peptides [11]. In 1980, Japanese researchers first isolated a small peptide from ginseng aqueous extract that inhibited adrenaline-induced lipolysis, hypothesizing its peptide nature based on amino acid composition [12]. Subsequent studies elucidated the sequence of the recombinant ginseng peptide as ETVEIIDSEGGGDA, a 14-amino acid peptide, and characterized its secondary structure [13]. Functional assays demonstrated its dual ability to suppress lipolysis and enhance cellular glucose uptake, positioning it as a promising hypoglycemic agent [14]. In type 2 diabetes mellitus (T2DM) mouse models, its hypoglycemic efficacy was confirmed, highlighting its potential to regulate systemic glucose homeostasis through these mechanisms [15]. This discovery has opened a new frontier in diabetes management, with small-molecule peptides from ginseng emerging as promising candidates for therapeutic development [16]. Despite the high therapeutic potential of ginseng-derived peptides, their industrial production remains constrained by conventional methods such as plant extraction and chemical synthesis [17]. These approaches suffer from significant drawbacks, including excessive costs, low purity, and environmental pollution due to toxic byproducts [18]. Recent advances in synthetic biology offer promising alternatives, with microbial biosynthesis emerging as a cost-effective and environmentally friendly method for producing bioactive peptides [19,20].

While prokaryotic systems (e.g., Escherichia *coli*, *Bacillus subtilis*) are widely used, their inability to perform post-translational modifications often leads to protein misfolding and inclusion body formation, complicating downstream purification [21,22].

In contrast, *Saccharomyces cerevisiae* (strain S288C), the first eukaryote with a fully sequenced genome (1996), stands out as a Generally Recognized as Safe (GRAS) organism with millennia of use in food fermentation (e.g., brewing, baking) [23,24]. At the beginning of the 21st century, most commercial yeast-derived products originated from *S. cerevisiae*, underscoring its industrial reliability [25]. Its well-characterized genetics, rapid growth in defined media, and compatibility with flexible induction strategies (e.g., galactose-inducible promoters) make it an ideal host for scalable, cost-effective production of bioactive peptides [26,27]. Moreover, the utilization of *Saccharomyces cerevisiae* as a host system effectively circumvents challenges associated with small peptides, including host protein interference, intracellular proteolytic degradation, and the requirement for proper folding, while simplifying downstream purification processes [28,29]. In addition, utilizing a multi-copy strategy effectively enhances gene expression. For instance, following the implementation of the multicopy tandem strategy, the expression levels of the recombinant ginseng hexapeptide increased by over 5-fold, while those of the beef flavor peptide improved by 2-fold [30].

Based on the aforementioned background, we proposed a genetically engineered method for producing recombinant ginseng peptide using *Saccharomyces cerevisiae* (BY4741) as the host. By employing a multicopy tandem expression strategy, we aim to enhance the expression and stability of the recombinant ginseng peptides, thereby increasing their yield and therapeutic potential. Furthermore, the biological activity of recombinant ginseng peptides was validated using an insulin-resistant fat cell model. By discussing the possible action mechanisms of recombinant ginseng peptides, we aim to demonstrate their potential as hypoglycemic peptides for application. This innovative approach addresses challenges in ginseng peptide production and offers new avenues for developing novel, safe, and effective treatments for diabetes.

## 2. Materials and Methods

### 2.1. Bacterial Strains, Plasmids, and Media

The bacterial strains and plasmids used in this study are listed in Table 1. *Escherichia coli* (*E. coli*) was grown in LB medium (lysogeny broth), while *Saccharomyces cerevisiae* (*S. cerevisiae*) was cultivated in YPD (yeast extract peptone dextrose) or SD-Ura (Synthetic Defined medium lacking uracil). Media were supplemented with ampicillin (100 μg/mL) or phosphate-buffered saline (PBS) as required by experimental protocols. Restriction enzymes BamHI and EcoRI, along with T4 DNA ligase, were obtained from Takara Biotechnology Co., Ltd. (Dalian, China). The positive control RS14 (recombinant ginseng tetradecapeptide) was chemically synthesized by Sangon Biotech Co., Ltd. (Shanghai, China).

### 2.2. Reagents and DNA Manipulation Techniques

The procedures for isolating and handling recombinant DNA followed established protocols. Tsingke Biotechnology Co., Ltd., located in Beijing, China, was responsible for carrying out DNA sequencing and synthesis, as detailed in Table 1. Essential molecular biology tools, such as restriction enzymes, were sourced from TaKaRa Biotechnology in Dalian, China. Tiangen Biotech, based in Beijing, China, provided the Prep mini plasmid kit. Furthermore, a kit for preparing sodium dodecyl sulfate-polyacrylamide gel electrophoresis (SDS-PAGE) was acquired from Sangon Biotech Co., Ltd. in Shanghai, China.

### 2.3. Molecular Construction and Transformation of S. cerevisiae

The digested fragments were ligated into the pYES2 vector, which had been linearized using BamH I and EcoR I, following the treatment of pET28-7RS14α vectors with BamH I and Xba I. The ligated product was then introduced into DH5α cells, and colonies containing the desired construct were identified and designated as pYES2-7RS14α. For yeast transformation, the Lithium Acetate Transformation Method was employed [31]. In this process, a suitable quantity of the positive transformants was mixed with competent *S. cerevisiae* cells and incubated at 37 °C with continuous shaking for 2 h. The mixture was subsequently plated onto an SD Agar Plate lacking URA (SD-Ura). Successful transformants were further confirmed through colony polymerase chain reaction (PCR) analysis [32].

### 2.4. Expression of 7RS14α in Flasks

Individual colonies of the genetically modified *S. cerevisiae* BY4741 strain were picked from SD-Ura agar plates and pre-cultivated for 24 h in 50 mL of SD medium at 30 °C. Subsequently, 2 mL of the freshly grown seed culture was transferred into 100 mL of YPD medium contained in 250 mL shake flasks and incubated at 30 °C until the optical density (OD) reached around 2 [33]. Following this, the culture was subjected to centrifugation, and the supernatant was removed. The resulting cell pellet was then re-suspended in YPG medium (Yeast Extract Peptone Galactose Medium), a step taken to eliminate any residual glucose that might disrupt the galactose induction process. The cells were induced to produce 7RS14α at 28 °C with agitation at 200 rpm, and the progression was monitored at various intervals.

### 2.5. Protein Detection and Purification

The target protein (7RS14α) was detected using either Western blot (WB) technique or Rapid Flag-tag Detection Kit (Detai Bioengineering, Nanjing, China). In the WB technique, after performing SDS-PAGE electrophoresis, the membrane was incubated with DYKDDDK tag Polyclonal antibody (Proteintech, Wuhan, China), followed by incubation with a corresponding secondary antibody (Rabbit). After thorough washing, the results were visualized through color development. When using the Flag detection test strips, 75 μL of the supernatant from the sample was applied to the loading area of the test strip, and the detection results were read after waiting for ten minutes.

The supernatant containing recombinant ginseng peptide and other heterologous proteins was obtained by centrifugation at 6000× *g* for 10 min at 4 °C. First, the supernatant was filtered through a 0.22 μm membrane, followed by purification using Anti-DYKDDDDK (Flag) Affinity Gel Anti-Flag (Yeasen Biotechnology, Shanghai, China) with an Affinity Chromatography Column (50 mL) provided by Sangon Biotech Co., Ltd. (Shanghai, China). Subsequently, the elution buffer was collected to obtain the recombinant ginseng protein (7RS14α). Finally, the protein was desalted and concentrated using a dialysis bag or a 15 kDa ultrafiltration tube. The target protein was detected by Western blot (WB). The purified 7RS14α was lyophilized and stored at −40 °C for future use [34].

### 2.6. Cell Culture, Treatment, and Activity Assay

The 3T3-L1 mouse embryonic fibroblasts were sourced from Shanghai Fuheng Biotechnology Co., Ltd. (Shanghai, China) and cultured in a humidified atmosphere with 5% carbon dioxide at 37 °C. The 3T3-L1 complete medium and the 3T3-L1 cell adipogenic induction differentiation and staining kit were also obtained from Shanghai Fuheng Biotechnology Co., Ltd. The trypsin (containing EDTA) used for digestion was purchased from Beijing Solarbio Science & Technology Co., Ltd. (Beijing, China).

Cell viability was assessed using the Cell Counting Kit-8 (CCK-8) assay, purchased from Abclone Scientific Kit (Wuhan, China), following the manufacturer’s protocol. Approximately 1 × 10^4^ cells were seeded into each well of a 96-well plate. The study was divided into six treatment groups: 0.1 mg/mL, 0.3 mg/mL, 0.5 mg/mL, 0.7 mg/mL, 0.9 mg/mL, and 1.1 mg/mL, along with a blank group and a control group. After 24 h, 10 μL of CCK-8 solution was added to each well, and the plates were incubated at 37 °C for 1–4 h. The absorbance of each well was measured at 450 nm.

### 2.7. Adipogenic Differentiation of Cells, Oil Red O Staining Observation, and Insulin-Resistant Cell Model Establishment

The 3T3-L1 cells were induced to differentiate into adipocytes following the protocol of the 3T3-L1 cell adipogenic induction differentiation and staining kit. After differentiation, the cells were fixed with paraformaldehyde (Beyotime, Shanghai, China) to preserve their morphology, and then stained with Oil Red O (Fuheng, Shanghai, China) to observe intracellular lipid droplets.

A 3T3-L1 insulin-resistant cell model was established by adding dexamethasone (Dex). After completing adipogenic induction and differentiation, the 3T3-L1 cells were cultured in growth medium containing 1 μmol·L^−1^ dexamethasone (Dex) for 24–120 h. Cell viability and glucose content were measured every 24 h to determine the optimal duration of dexamethasone treatment [35].

### 2.8. The Insulin Synergistic Effect on the Insulin-Resistant Cell Model Cells Was Assessed

After inducing differentiation in six-well plates at a density of 5 × 10^5^ cells per well or in 48-well plates at a density of 5 × 10^4^ cells per well, the cells were divided into two control groups, two model groups, and two experimental groups. An additional set of control and model groups included insulin (10^−7^ mmol/L^−1^). The experimental groups consisted of model cells treated with 0.1 mg/mL of the target protein and model cells treated with both insulin (10^−7^ mmol/L^−1^) and 0.1 mg/mL of the target protein. After 24 h of incubation, the glucose concentration in the supernatant of the 48-well plate cells was measured using a glucose content detection kit, while the triglyceride content in the cell lysates of the six-well plate cells was determined using a triglyceride detection kit. All reagents mentioned above were obtained from the Nanjing Jiancheng Bioengineering Institute.

## 3. Results

### 3.1. Molecular Engineering, Heterologous Expression, and Purification of Recombinant Ginseng Polypeptide

**Figure 1:**
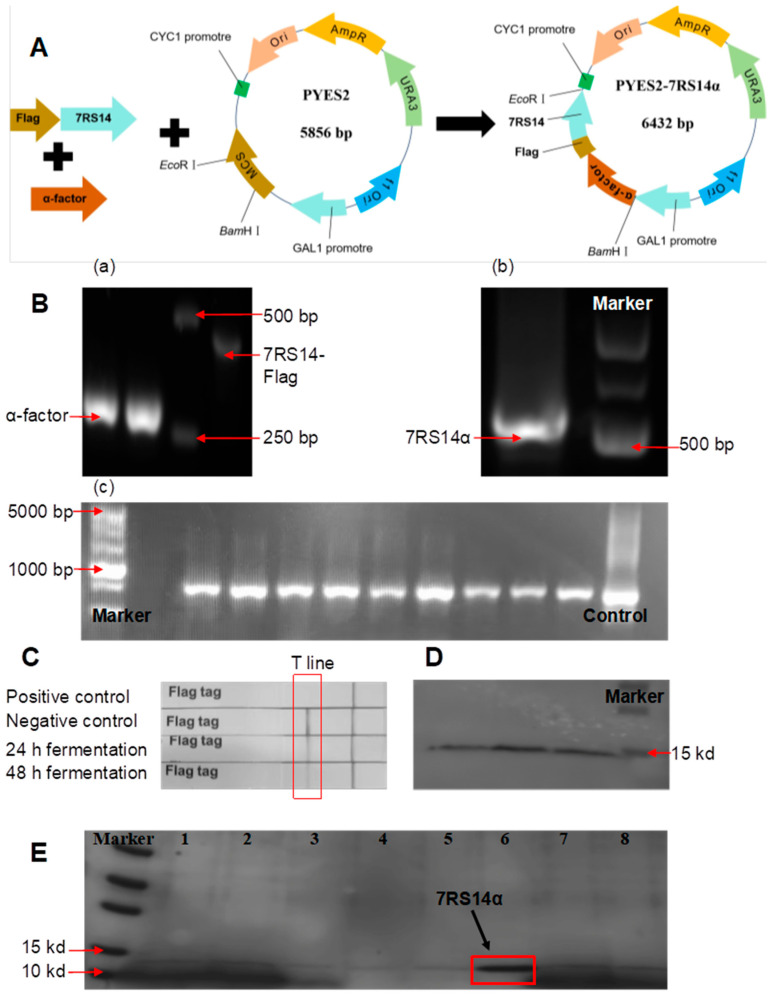
Construction, transformation, protein expression and purification of engineered strains. (**A**) Graphical representation of the key steps in plasmid engineering. (**B**) Construction and validation of engineered strains: (**a**) the cloning of α-factor fragment and 7RS14-Flag fragment; (**b**) PCR validation of recombinant plasmids; (**c**) colony PCR verification of BY4741-pYES2-7RS14α. (**C**) Rapid detection of 7RS14α expression using colloidal gold-based FLAG-tag immunoassay kit. (**D**) Western blot analysis of 7RS14a protein expression. (**E**) Purification of 7RS14a using FLAG affinity chromatography (Lane 1–2: Flow-through fraction from fermentation broth, Lane 3: wash buffer fraction, Lane 4–6: elution fractions with varying FLAG peptide concentrations (0.1mg/mL,0.3 mg/mL,0.5 mg/mL), Lane 7–8: regeneration wash buffer fraction).

The PYES2 plasmid, a commercially available shuttle plasmid containing a GAL1 promoter, was selected to induce the expression of recombinant ginseng peptides upon switching the carbon source to galactose. The 14-amino acid ginseng peptide sequence (ETVEIIDSEGGGDA) was designed to be concatenated head-to-tail seven times. Additionally, the truncated α-factor secretion signal by removing its C-terminal four amino acids was used to ensure extracellular secretion of the target peptide without residual amino acids, thereby minimizing potential interference with peptide bioactivity. The FLAG epitope tag was inserted at the 5′-end of the recombinant peptide sequence to enable rapid detection and facilitate high-purity isolation of the target protein. The construct designated plasmid PYES2-7RS14α is shown in Figure 1A.

The 7RS14-Flag peptide gene sequence fused with the FLAG tag was chemically synthesized and amplified via PCR, as shown in Figure 1B(a), confirming successful amplification of both fragments. These fragments were subsequently ligated into the PYES2 plasmid using homologous recombination. The recombinant plasmid was verified by PCR with primers specific to the PYES2 plasmid’s sequencing sites (Figure 1B(b)). Agarose gel electrophoresis revealed that the recombinant fragment migrated slightly larger than 500 bp, consistent with its theoretical size (576 bp) (Figure 1B(b)). The excised band was subjected to Sanger sequencing, which confirmed 100% identity with the designed nucleotide sequence, validating the successful construction of the recombinant fragment. The recombinant plasmid was then transformed into *Saccharomyces cerevisiae* obtain recombinant yeast cells. The gene of the recombinant ginseng peptide has been successfully transferred into the *Saccharomyces cerevisiae* cells (BY4741-PYES2-7RD14α), which is proved by yeast colony PCR (Figure 1B(c)).

The engineered BY4741-pYES2-7RS14α strain was induced in shake flasks for 48 h. Supernatants from 24 h and 48 h fermentation cultures were collected after centrifugation and analyzed for target protein expression using lateral flow FLAG-tag detection. A red band at the test line (T line) indicates the absence of FLAG-tagged protein in the sample. Figure 1C demonstrates the presence of FLAG-tagged protein in both 24 h and 48 h supernatants, confirming successful expression of the target peptide.

Western blot (WB) analysis (Figure 1E) further validated the identity of the peptide, showing a distinct band within the 10–15 kDa range, aligning with the electrophoretic results in Figure 1D. For competitive elution, three concentrations of FLAG peptide solutions (0.1 mg/mL, 0.3 mg/mL, and 0.5 mg/mL) were tested. As shown in Figure 1E, the 0.5 mg/mL FLAG peptide solution achieved optimal elution efficiency, yielding a peptide band between 10 and 15 kDa—consistent with the theoretical size of the recombinant ginseng peptide (10 kDa). ImageJ (v1.8.0) analysis confirmed >95% purity. Approximately 20 mg/L of recombinant ginseng peptide was obtained per liter of fermentation in a 500 mL flask broth after desalting and lyophilization, enabling subsequent functional studies.

### 3.2. Characterization of Recombinant Ginseng Polypeptides

**Figure 2:**
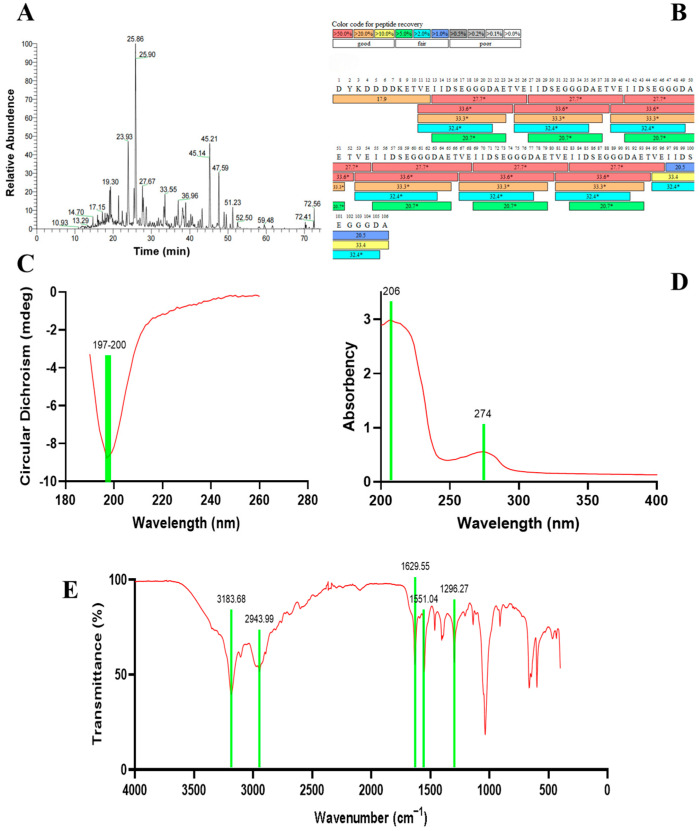
Systematic characterization of bioactive properties in recombinant ginseng-derived polypeptides. (**A**) Total I on chromatogram (TIC) analysis. (**B**) Sequence coverage map analysis. * indicates peptide not unique. (**C**) Circular dichroism (CD) spectroscopic analysis. (**D**) Ultraviolet-visible (UV–Vis) absorption spectroscopic analysis. (**E**) Fourier-transform infrared (FTIR) spectroscopic analysis.

Figure 2A illustrates the peptide profile of the purified recombinant ginseng polypeptide following in-gel digestion and LC-MS/MS analysis, demonstrating efficient enzymatic cleavage. Further validation using Thermo BioPharma Finder (5.0) software for mass spectrometry database searching revealed a 100% sequence coverage alignment between the recombinant polypeptide produced by the engineered strain and the theoretical amino acid sequence (Figure 2B). These results confirm the feasibility of the *Saccharomyces cerevisiae* engineering system for successful expression of the recombinant ginseng polypeptide.

The far-UV circular dichroism (CD) spectrum of the recombinant ginseng polypeptide (Figure 2C) provides insights into the arrangement of peptide bonds, while secondary structure analysis using CDNN (11.2) software revealed the proportions of α-helix, β-sheet, β-turn, and random coil configurations (Table 2). The ultraviolet (UV) absorption spectrum of the recombinant polypeptide (Figure 2D) exhibited characteristic peaks at 206 nm and 274 nm, which are indicative of peptide bond and aromatic residue contributions, respectively, and can be utilized for quantitative determination. Fourier-transform infrared (FTIR) spectroscopy (Figure 2E) further confirmed structural features, namely absorption bands at 3183 cm^−1^ (N–H and O–H stretching vibrations), 2943 cm^−1^ (C–H stretching vibrations), and 1629 cm^−1^ (β-sheet structure, consistent with CD analysis). Additional peaks at 1551 cm^−1^ (amide II band, C–N stretching and N–H bending) and 1296 cm^−1^ (amide III band) further validated the polypeptide’s structural integrity.

### 3.3. Evaluation of the Biological Activity of Recombinant Ginseng Tetradecapeptide

**Figure 3:**
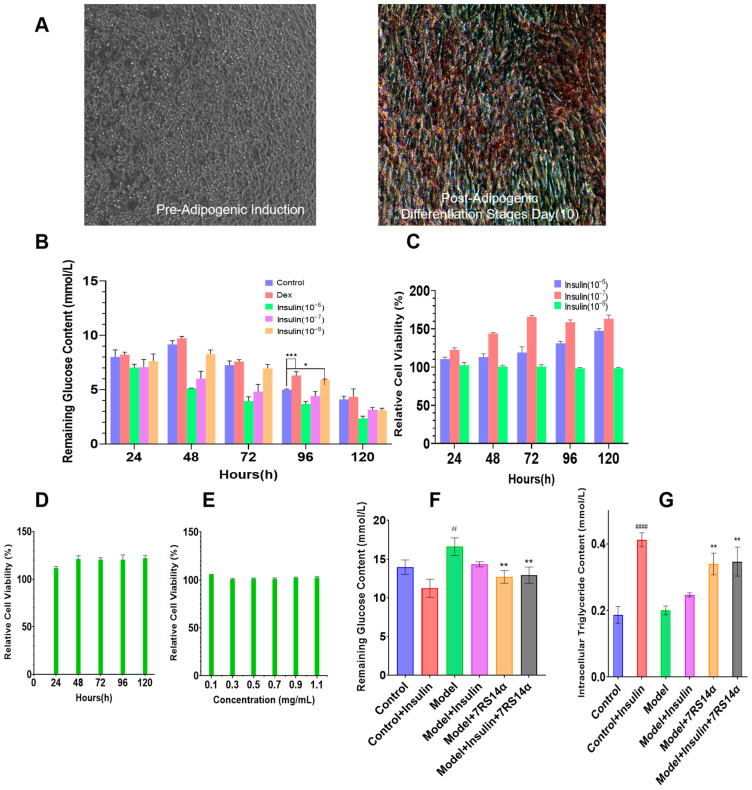
Effects of 7RS14α on insulin-resistant model cells. (**A**) Microscopic evaluation of 3T3-L1 adipocyte differentiation: comparative analysis of pre-and post-adipogenic (Day 10) induction with oil red O staining (10×, red deposits indicate intracellular lipid accumulation). (**B**) Effects of treatment duration and drug type on the establishment of insulin-resistant cell models (1 μmol/L Dex and 10^−6^ mmol/L, 10^−7^ mmol/L, 10^−8^ mmol/L Insulin). (**C**) The changes in cell viability of differentiated 3T3-L1 adipocytes, treated with three concentrations of insulin for 120 h (10^−6^ mmol/L, 10^−7^ mmol/L, 10^−8^ mmol/L). (**D**) The changes in cell viability of differentiated 3T3-L1 adipocytes treated with 1 μmol/L Dex for 120 h. (**E**) Dose-dependent effects of recombinant ginseng polypeptides on cellular viability in differentiated 3T3-L1 adipocytes. (**F**) The impact of 7RS14α on glucose uptake in murine adipocytes. (**G**) The impact of 7RS14α on triglyceride content in murine adipocytes. * *p* < 0.05 versus model group, ** *p* < 0.01 versus model group, and *** *p* < 0.001 versus model group, # *p* < 0.05 versus control group, #### *p* < 0.0001 versus control group.

The earliest identified activity of ginseng tetradecapeptide was its antilipolytic effect on adipocytes, and subsequent studies further discovered its glucose absorption-promoting activity. We believe that recombinant ginseng peptides are likely to also exert activity on adipocytes. Therefore, to evaluate the glucose absorption-promoting activity of recombinant ginseng tetradecapeptide, we attempted to use 3T3-L1 cells, which are the most commonly used cells for studying adipocyte growth and metabolism. First, 3T3-L1 cells were induced to differentiate into adipocytes using a differentiation induction kit. The 3T3-L1 cell line, derived from mouse embryonic fibroblasts, is a preadipocyte line with specific adipogenic differentiation potential and is widely used as a model for studying lipid metabolism. As shown in Figure 3A, significant lipid droplets were observed in cells after 10 days of induction, confirming successful differentiation of 3T3-L1 cells into adipocytes. Next, dexamethasone (Dex) and varying concentrations of insulin were employed to establish the insulin-resistant model. The results shown in Figure 3B reveal that adipocytes treated with 1 μmol/L Dex and 10^−8^ mmol/L insulin exhibited significantly reduced glucose uptake compared to the control group at 96 h. Notably, Dex-treated adipocytes showed more pronounced differences in glucose uptake relative to controls. Therefore, 1 μmol/L Dex treatment for 96 h was selected to construct the insulin-resistant cell model.

To further confirm that the reduction in cellular glucose uptake was not due to compromised cell viability, the CCK-8 assay was performed on cells treated with varying insulin concentrations and 1 μmol/L Dex. As shown in Figure 3C,D, cell viability was higher in treated cells compared to untreated controls, confirming that the decreased glucose uptake was independent of cell survival. Subsequently, to determine the safe dosage of the recombinant ginseng peptide, mouse adipocytes were cultured in media supplemented with 0.1 mg/mL, 0.3 mg/mL, 0.5 mg/mL, 0.7 mg/mL, 0.9 mg/mL, or 1.1 mg/mL recombinant peptide, and cell viability was assessed. A concentration that neither affected cell growth nor impairs the effect of promoting glucose absorption was prioritized. The results shown in Figure 3E indicate that 0.1 mg/mL recombinant peptide exhibited the most favorable impact on cell viability, and this concentration was selected for subsequent experiments.

Figure 3F compares the effects of recombinant ginseng peptide and insulin on glucose uptake in insulin-resistant model cells. Insulin treatment significantly increased glucose uptake in normal mouse adipocytes, confirming their insulin sensitivity. In contrast, insulin-resistant model cells exhibited reduced basal glucose uptake, which remained elevated even after insulin treatment, indicating insulin resistance. Strikingly, supplementation with recombinant ginseng peptide further increased glucose uptake in the model cells, achieving levels nearly comparable to insulin-treated normal cells. These findings highlight the potential of recombinant ginseng peptides to be applied as hypoglycemic peptides. Concurrently, Figure 3G demonstrates that insulin treatment significantly increased intracellular triglyceride (TG) content in normal adipocytes, whereas model cells showed no response to insulin. However, recombinant ginseng peptide supplementation elevated TG content in model cells, suggesting insulin-mimetic activity and highlighting its potential to modulate insulin-resistant cells.

**Figure 4:**
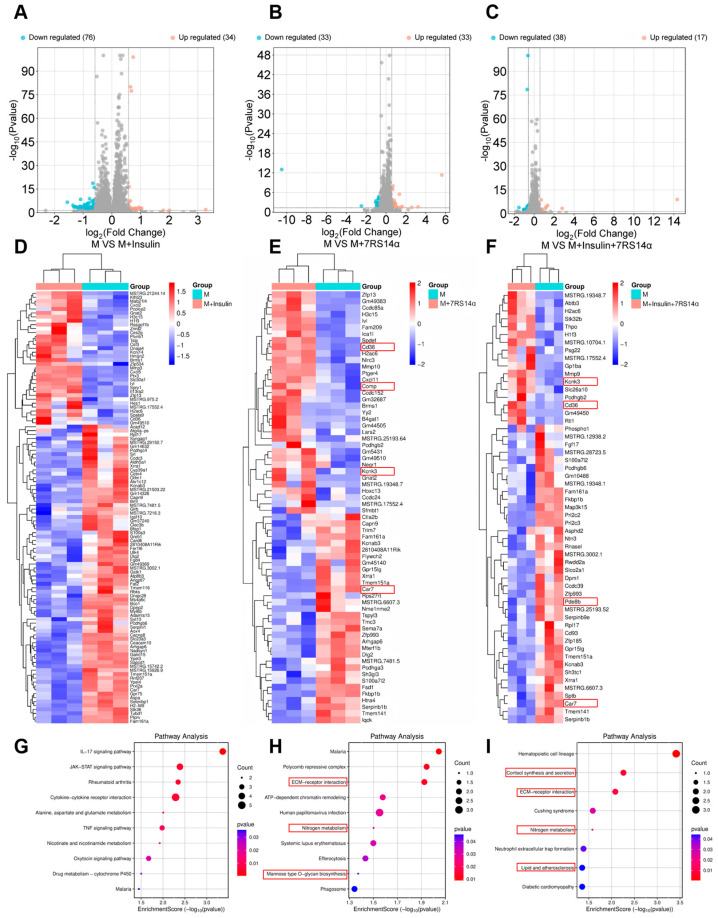
Transcriptomic analysis of 7RS14α effects on insulin-resistant adipocytes (*p* < 0.05). (**A**–**C**): Volcano plots of differentially expressed genes (DEGs) for comparisons: M vs. M + insulin, M vs. M + 7RS14α, and M vs. M + insulin + 7RS14α. (**D**–**F**): Hierarchical clustering heatmaps based on differentially expressed genes (DEGs) for comparisons: M vs. M + insulin, M vs. M + 7RS14α, and M vs. M + insulin + 7RS14α. (**G**–**I**): Enriched pathway maps based on differentially expressed genes (DEGs) for comparisons: M vs. M + insulin, M vs. M + 7RS14α, and M vs. M + insulin + 7RS14α.

To further investigate the mechanism of the recombinant ginseng peptide, eukaryotic reference-based transcriptomic analysis was performed on insulin-resistant model cells treated with either the recombinant peptide or insulin. Four experimental groups were analyzed: insulin-resistant model cells (M), M treated with insulin (10^−7^ mmol/L) (M + insulin), M treated with 7RS14α (0.1 mg/mL) (M + 7RS14α), and M treated with both insulin and 7RS14α (M + insulin + 7RS14α), with three biological replicates per group. Volcano plots of differentially expressed genes (DEGs) for comparisons between the M group and the other three groups (M + insulin, M + 7RS14α, M + insulin + 7RS14α) are shown in Figure 4A–C. The x-axis represents log_2_FoldChange (log_2_FC), indicating the fold change in gene expression between groups, and the y-axis represents −log_10_ *p*-value, reflecting the statistical significance of expression differences. Red and blue dots denote upregulated and downregulated genes, respectively. The limited number of DEGs across all comparisons likely reflects insulin-resistance-induced transcriptional stability. Hierarchical clustering heatmaps of these DEGs are presented in Figure 4D–F. Genes with consistent expression trends in both the M + 7RS14α and M + insulin + 7RS14α groups—Kcnk3, CD36, and Car7—were identified as potential key mediators of the recombinant peptide’s bioactivity.

The Kcnk3 gene, a member of the two-pore potassium channel (K2P) family, regulates cellular membrane potential and excitability, likely influencing ion homeostasis and signaling pathways in mouse adipocytes to modulate metabolic activity, differentiation, and function. Upregulation of Kcnk3 alters the electrophysiological properties of adipocytes, potentially affecting responses to external signals [36]. Concurrently, membrane potential shifts may impact the activity of metabolic enzymes and receptor signaling, such as lipid metabolism-associated enzymes and insulin receptor pathways, suggesting that 7RS14α modulates adipocyte metabolism and energy balance through these mechanisms. The CD36 gene, involved in fatty acid uptake, lipid metabolism, and signaling, may regulate adipocyte energy homeostasis by modulating fatty acid transport, insulin signaling, and inflammation/immune pathways [37]. The Car7 gene catalyzes the conversion of CO_2_ and H_2_O into HCO_3_− and H+, influencing intracellular pH, which is linked to enzyme activity, signal transduction, and oxidative stress. Additionally, HCO_3_− serves as a carbon source for fatty acid synthesis. In mouse adipocytes, Car7 may affect cellular metabolism and energy balance by altering pH dynamics and fatty acid synthesis [38].

KEGG pathway enrichment analysis of differentially expressed genes across the three experimental groups is shown in Figure 4G–I. The M + insulin group exhibited significant activation of the IL-17 and JAK-STAT signaling pathways, likely attributable to insulin resistance. In the M + 7RS14α group, enriched pathways related to metabolism included ECM–Receptor Interaction, Nitrogen Metabolism, and Mannose Type O-Glycan Biosynthesis. Upregulation of CD36 in the ECM–Receptor Interaction pathway suggests activation of cell surface receptors involved in lipid metabolism, potentially enhancing lipid synthesis [39]. Downregulation of Car7 in the Nitrogen Metabolism pathway implies reduced HCO_3_− availability, dampening nitrogen metabolism [40]. Upregulation of B4gat1 in the Mannose Type O-Glycan Biosynthesis pathway indicates enhanced O-glycosylation of glycoproteins, which may strengthen insulin receptor O-glycosylation to potentiate insulin signaling [41].

## 4. Discussion

In the M + insulin + 7RS14α group, key metabolic pathways included cortisol synthesis and secretion, ECM–receptor interaction, nitrogen metabolism, and lipid and atherosclerosis. The ECM–receptor interaction and nitrogen metabolism pathways mirrored those in the M + 7RS14α group. In the cortisol synthesis and secretion pathway, upregulation of Kcnk3 may alter membrane potential to reduce local cortisol utilization, thereby suppressing triglyceride breakdown, while downregulation of Pde8b could elevate cAMP levels, increasing cortisol availability to accelerate lipolysis [42]. Upregulation of CD36 in the Lipid and Atherosclerosis pathway suggests enhanced fatty acid transport and lipid synthesis [43]. Collectively, these findings indicate that 7RS14α modulates energy metabolism in mouse adipocytes by targeting lipid metabolism pathways and insulin signaling cascades.

Diabetes mellitus, a chronic metabolic disorder characterized by hyperglycemia, ranks as the third most prevalent noncommunicable disease globally, following cancer and cardiovascular diseases, posing substantial public health challenges. Current clinical management relies on therapeutics including insulin analogs, insulin secretagogues, α-glucosidase inhibitors, and insulin sensitizers. Existing α-glucosidase inhibitors are primarily derived from microbial metabolites, natural extracts, and synthetic/semi-synthetic compounds [44]. Despite therapeutic advances, suboptimal long-term glycemic control persists in many patients. Concurrently, growing interest in complementary and alternative medicine (CAM), particularly among diabetic populations, established botanical interventions as promising adjunctive therapies [45]. Among botanical CAMs, ginseng has demonstrated notable potential in the management of type 2 diabetes mellitus (T2DM) [46]. In this study, we developed a biosynthetic approach to produce a recombinant ginseng polypeptide, analyzed its secondary structure, validated its glucose-regulatory efficacy in vitro using adipocyte models, and investigated its molecular mechanisms of action. These findings advance the development of this recombinant polypeptide as a novel antidiabetic agent.

The recombinant ginseng peptide yield via *Saccharomyces cerevisiae* expression remains suboptimal, with ~20 mg/L obtained from fermentation broth. However, molecular-level optimizations (e.g., codon usage bias adjustment, promoter engineering) combined with fermentation process enhancements (e.g., targeted nutrient supplementation, induction timing control) could substantially improve productivity. *S. cerevisiae* as a host ensures exceptional biosafety and enables direct application of fermentation products, fulfilling compliance requirements for therapeutic/nutraceutical use. Critically, the recombinant polypeptide exhibited 100% sequence identity with the native ginseng tetradecapeptide, confirming fidelity in expression. Secondary structure analysis provided insights into its spatial conformation, establishing a foundation for future structure-activity relationship studies. Functionally, the recombinant polypeptide significantly reduced glucose uptake in insulin-resistant adipocytes, demonstrating mechanisms conducive to glucose stabilization. Transcriptomic profiling further revealed its modulation of lipid metabolic enzymes and insulin signaling pathways, consistent with observed phenotypic effects. These findings collectively validate the therapeutic potential of this recombinant polypeptide for diabetes management while delineating molecular targets for mechanistic exploration and translational development.

Additionally, despite the relatively low yield of recombinant ginseng tetradecapeptide (7RS14α), its production via microbial synthesis demonstrates superior cost-effectiveness compared to plant extraction and chemical synthesis. The material cost for 1 L of fermentation broth is approximately CNY 50, with affinity gel purification adding around CNY 3500, yielding 10–20 mg of recombinant peptide. In contrast, plant extraction requires 1 kg of ginseng powder (costing CNY ~2000) to obtain merely 2 mg of the peptide, alongside complex purification steps involving organic solvents, prolonged timelines, and low overall efficiency. Chemical synthesis, while capable of producing 20 mg of the peptide at a cost of CNY ~2000, involves labor-intensive stepwise amino acid coupling (Table 3). Both plant extraction and chemical synthesis face challenges of significant environmental pollution and procedural complexity. Given the high safety profile of *Saccharomyces cerevisiae*, future studies could explore direct use of fermentation broth to streamline purification and further reduce costs, enhancing the economic viability of this biosynthetic approach.

Given the extremely high safety of *Saccharomyces cerevisiae*, fermented products containing heterologous recombinant proteins obtained using *S. cerevisiae* as a host have the potential to be directly used as products or raw materials. Whether such fermented products can be directly used as products or raw materials is mainly determined by the laws and regulations of different countries and regions. For example, China currently does not allow fermented products produced by genetically engineered strains to be directly used in the food industry. However, fermented products produced by genetically engineered strains can be used in the feed industry after routine industry testing, as well as additional sterilization steps and PCR detection (to ensure no exogenous gene fragments remain). Direct use of fermented products as products or raw materials offers significant economic advantages due to the elimination of separation and purification steps. The genetically engineered strain BY4741-pYES2-7RS14α constructed in this study has the potential for its fermented products to be directly used as products or raw materials. However, to further scale up production, continuous optimization of the medium formulation and fermentation method is required.

## Figures and Tables

**Table 1:** Strains and plasmids used in this work.

Strain or Plasmid	Description	Source/ Reference
BY4741	*Saccharomyces cerevisiae* S288C-derivative laboratory strain, MATa his3Δ1 leu2Δ0 met15Δ0 ura3Δ0	lab stock
DH5α	*Escherichia coli* cloning strain	lab stock
pYES2	plasmid *E. coli*–*S. cerevisiae* shuttle vector, Amp^R^ for *E. coli*	lab stock
pET28-7RS14	*E. coli* vector, Amp^R^, pET28 carrying seven replicate fragments of ginseng tetradecapeptide and FLAG tag.	Sagon

Amp^R^, ampicillin resistance; Sagon, Sangon Biotech (Shanghai) Co., Ltd. (Shanghai, China).

**Table 2:** The secondary structure proportions of recombinant ginseng tetradecapeptide.

Name	Protein Secondary Structure				
	Helix	Beta-Pleated Sheet		Beta-Turn	Rndm. Coil
		Antiparallel	Parallel		
7RS14α	8.5%	39.5%	3.1%	19.6%	29.4%

**Table 3:** Comparison of microbial synthesis, plant extraction and chemical synthesis.

Name	Microbial Synthesis	Plant Extraction	Chemical Synthesis
material cost	CNY 50	CNY 2000	CNY 2000
time	a week	more than a week	more than a week
yield	10–20 mg recombinant ginseng peptide	2 mg ginseng peptide	20 mg ginseng peptide

## Data Availability

The original contributions presented in the study are included in the article; further inquiries can be directed to the corresponding author.

## Abbreviations

The following abbreviations are used in this manuscript:

CAM	Complementary and Alternative Medicine
T2DM	Type 2 Diabetes Mellitus
GRAS	Generally Recognized as Safe
YPD	Yeast Extract Peptone Dextrose Medium
SD-Ura	Synthetic Dropout Medium-Uracil
PBS	Phosphate-Buffered Saline
PCR	Polymerase Chain Reaction
SDS-PAGE	Sodium Dodecyl Sulfate-PolyAcrylamide Gel Electrophoresis
OD	Optical Density
LC-MS	Liquid Chromatograph Mass Spectrometer
WB	Western Blot
CD	Circular Dichroism Spectroscopy
CDNN	Cluster-based Discriminative Neural Networks
UV	Ultraviolet
FTIR	Fourier-Transform Infrared Spectroscopy
TIC	Total I on Chromatogram
Dex	Dexamethasone
CCK-8	Cell Counting Kit-8
TG	Triglyceride
FC	FoldChange
DEGs	Differentially Expressed Genes
KEGG	Kyoto Encyclopedia of Genes and Genomes
CNY	Chinese Yuan
